# The role of an active surveillance strategy of targeting household and neighborhood  contacts related to leprosy cases released from treatment in a low-endemic area of China

**DOI:** 10.1371/journal.pntd.0008563

**Published:** 2020-08-14

**Authors:** Na Wang, Tongsheng Chu, Furong Li, Zhenzhen Wang, Dianchang Liu, Mingfei Chen, Honglei Wang, Guiye Niu, Dan Liu, Mingkai Zhang, Yuanyuan Xu, Yan Zhang, Jinghui Li, Zhen Li, Jiabao You, Liguo Mao, Huaizhang Li, Yongjin Chen, Hong Liu, Furen Zhang

**Affiliations:** 1 Shandong Provincial Hospital for Skin Diseases & Shandong Provincial Institute of Dermatology and Venereology, Shandong First Medical University & Shandong Academy of Medical Sciences, Jinan, Shandong, China; 2 Shandong Provincial Key Lab for Dermatovenereology, Jinan, Shandong, China; 3 Shandong Provincial Medical Center for Dermatovenereology, Jinan, Shandong, China; 4 Jining City Dermatology Hospital Prevention and Treatment, Jining, Shandong, China; 5 Zaozhuang Dermatology Hospital Prevention and Treatment, Zaozhuang, Shandong, China; 6 Linyi Dermatology Hospital, Linyi, Shandong, China; Federal University of Ceará, Fortaleza, Brazil, BRAZIL

## Abstract

**Objective:**

Early diagnosis remains the primary goal for leprosy management programs. This study aims to determine whether active surveillance of patients with leprosy and their contact individuals increased identification of latent leprosy cases in the low-endemic areas.

**Methods:**

This cross-sectional survey was carried out between October 2014 and August 2016 in 21 counties throughout Shandong Province. The survey was conducted among patients with leprosy released from treatment (RFT) and their contacts from both household and neighbors.

**Results:**

A total of 2,210 RFT patients and 9,742 contacts comprising 7877 household contacts (HHCs), including 5,844 genetic related family members (GRFMs) and 2033 non-genetic related family members and 1,865 contacts living in neighboring houses (neighbor contacts, NCs), were recruited. Among identified individuals, one relapsed and 13 were newly diagnosed, giving a detection rate of 0.12%, corresponding to 120 times the passive case detection rate. Detection rates were similar for HHCs and NCs (0.114% vs. 0.214%, P = 0.287). Analysis of the family history of leprosy patients revealed clustering of newly diagnosed cases and association with residential coordinates of previously-diagnosed multibacillary leprosy cases.

**Conclusion:**

Active case-finding programs are feasible and contributes to early case detection by tracking HHCs and NCs in low-endemic areas.

## Author summary

Leprosy has been eliminated as a public health problem in 1994 in Shandong Province. However, the district continues to report a relatively high number of cases of leprosy infection involving deformity. Several studies have shown that individuals in contact with people infected with leprosy are at high risk of developing the disease. Subclinical infections among such individuals are important in the chain of *M*. *leprae* transmission. Some hyperendemic areas show growing interest in active case finding (ACF). Recent data from the World Health Organization (WHO) show that high rates of relapsed patients, grade 2 disability (G2D) since 2011, and the extensive family history of leprosy among people in Shandong Province P.R. China, indicate a need to reconsider the current approach to leprosy prevention. Active case finding was conducted in 21 counties of Shandong Province among patients with leprosy released from treatment (RFT) and their contacts. We achieved a detection rate of 0.12%, which was much higher than the rate for passive case finding. Our ACF program confirmed the need to implement this strategy among families and neighbors of RFT patients in historically high-endemic areas of leprosy. The program could reduce the risk of G2D by facilitating early detection and treatment, thereby reducing the disease burden.

## Introduction

Leprosy is a chronic infectious disease caused by *Mycobacterium leprae*, an obligate intracellular pathogen. As one of the oldest recorded diseases [1], leprosy has been associated throughout history with fear, prejudice, and social stigma. Deformity and disability lead to negative feelings and discrimination [2]. According to World Health Organization (WHO) data, the proportion of grade 2 disability (G2D) cases among newly diagnosed leprosy patients was 5.97% globally and 20.3% in China [3]. With a high G2D rate, leprosy remains an important public health problem in many parts of the world [4].

Early detection and timely treatment of leprosy are key for interrupting transmission and preventing physical and social complications and thus reducing disease burden [5]. However, as a relatively rare skin disease, early identification and diagnosis of leprosy patients remains a challenge, especially in low-endemic areas. Studies show that individuals living with an untreated leprosy patient, especially household contacts (HHCs), have the highest risk of developing clinical disease, at 6–28% [6–8]. Risk of disease among HHCs further increases with close contact with a patient with multibacillary (MB) leprosy before diagnosis or seropositivity to *M*. *leprae*-specific antigens, especially in the absence of Bacille Calmette-Guerin (BCG) vaccination [9]. Higher incidence of new leprosy cases among HHCs is reported, such as 50 per 100,000 in Bangladesh and 1.5 per 100,000 in Thailand [7,10]. Hence, a strategy based on detection of close contacts (including HHCs and neighbor contacts [NCs]) of patients with leprosy such as an active case finding (ACF) program may be an effective tool for early detection.

In Brazil, ACF programs have been used to reduce transmission of leprosy by identifying people with active or subclinical infections [6]. These programs offer better diagnostic facilities, particularly for asymptomatic and smear-negative patients, which could mitigate disability, accelerate diagnosis and treatment, and interrupt transmission in high endemic areas [6]. Chen et al. performed a retrospective study using a questionnaire-based survey to assess the need for an ACF program for HHCs of index patients in the low-endemic area of Shandong Province, China [11]. Only a small proportion of patients (13.3% in a 5-year follow-up and 11.1% in a 10-year follow-up) were HHCs. This result led the authors to conclude that there is a need for other approaches to leprosy detection. In 2006, the WHO Global Strategy Plan stated that the active search for leprosy cases should be discontinued. The primary focus switched to passive case detection only [12].

Therefore, tracking and screening contacts of newly diagnosed patients with leprosy was atrophied in Shandong Province in 2006. The early detection of new cases mainly relies on spontaneous presentation of patients to the health system for clinical examination. The BCG vaccination has been routinely used, however, there still have less than 30 newly-detected cases per year in Shandong province with a population of more than 10 million (the leprosy prevalence is less than 1/300 000), while the G2D rate in Shandong was 42.9% according to data from the National Leprosy Management Information System (LEPMIS) of Shandong Province from 2006 to 2016. This rate was much higher than the 20% previously reported by the WHO [13]. The data showed that 17.8% of all patients with new cases of leprosy had G2D and 47.7% of patients with G2D had a family history of leprosy. Thus there is evidence for both ongoing transmission (i.e. HHC) and considerable delay until diagnosis (i.e. G2D). Since leprosy has initially painless and inconspicuous symptoms which can easily be mistaken for other skin disorders, patients tend to overlook symptoms and do not seek medical treatment at an early stage. Consequently, leprosy is often ignored or diagnosed late, and some newly-diagnosed patients suffer from severe deformities. The current control status of leprosy therefore indicates a need to reconsider the current approach to prevention, and we performed a cross-sectional study to determine whether restarting active surveillance of HHCs and NCs can increase identification of early leprosy cases in low-endemic areas.

## Methods

The survey was conducted from October 2014 through August 2016 in 21 counties of Shandong province selected based on the historical data of leprosy patients from the National LEPMIS, which accounted for 80% of RFT patients in the history of Shandong Province, China (maps in S1 Fig). All participants were RFT patients registered in LEPMIS as index patients, or were HHCs, or NCs, defined as neighbors living within a radius of about 200 meters around an index patient. This distance was empirically established, based on the work of Barreto et al [14]. The tracing status and steps of index patients and their contacts coincided with Fürst et al [15].

To strengthen the awareness and avoid absenteeism, the RFT patients and their contacts were informed by the local health agency staff of leprosy control and the exact time of the visit a few days prior to the active surveillance. All the RFT patients were familiarized with the aims, benefits, procedures, and potential risks of the intervention, and written informed consent to disclose the disease status to household members and neighbors. With consent, RFT patients and their contacts were referred to local centers for leprosy prevention and control. Home visits were performed for older patients. All the participants were informed about the program, procedures, the possible consequences of the different screening outcomes. Furthermore, a well-maintained national leprosy database (LEPMIS) was essential for this survey.

A team of four professional leprosy-control physicians, six postgraduate medical students, and three social workers performed data collection and physical examinations for all participants. Before the survey, training was conducted for all the team members, explaining the purpose of the ACF, as well as the ACF procedures, how to complete the questionnaire and how to register the relationship between the contacts and neighbors. All the registered RFT patients were visited and re-examined to confirm their leprosy status. A verbally-administered questionnaire and one-page self-image form were used to collect demographic and clinical information. All individuals were given physical and dermatological examinations, to identify common signs of leprosy lesions such as hypo-pigmented areas with loss of sensation, scaly or reddish-bordered skin lesions, and other cardinal signs of leprosy, and particularly to note any superficial nerves of the body which were swollen or painful. Any deformities were documented. Patients with lesions suggestive of leprosy were referred to the Shandong Provincial Institute of Dermatology and Venereology (China) for further examination, including the slit skin smear test and skin biopsy. Diagnosis and clinical classification were based on criteria of Ridley and Jopling [16], and the diagnosis was confirmed by quantitative polymerase chain reaction of *RLEP* and *groEL* based on nucleic acid detection for leprosy diagnosis, using an ABI Step One Plus real-time PCR system. After confirmation of diagnoses, new patients were treated using multidrug therapy, as recommended by the WHO [17]. For NCs without any signs of leprosy disease, only summary data were obtained (e.g. number of contacts screened).

### Maps of copyright

Details about the location of the counties were marked in the map. The maps were created using an open source map (http://landsatlook.usgs.gov/) without copyright. The maps were under the Creative Commons Attribution License which permits unrestricted use, distribution, and reproduction in any medium, provided the original work is properly cited.

### Data analysis

**Fig 1:**
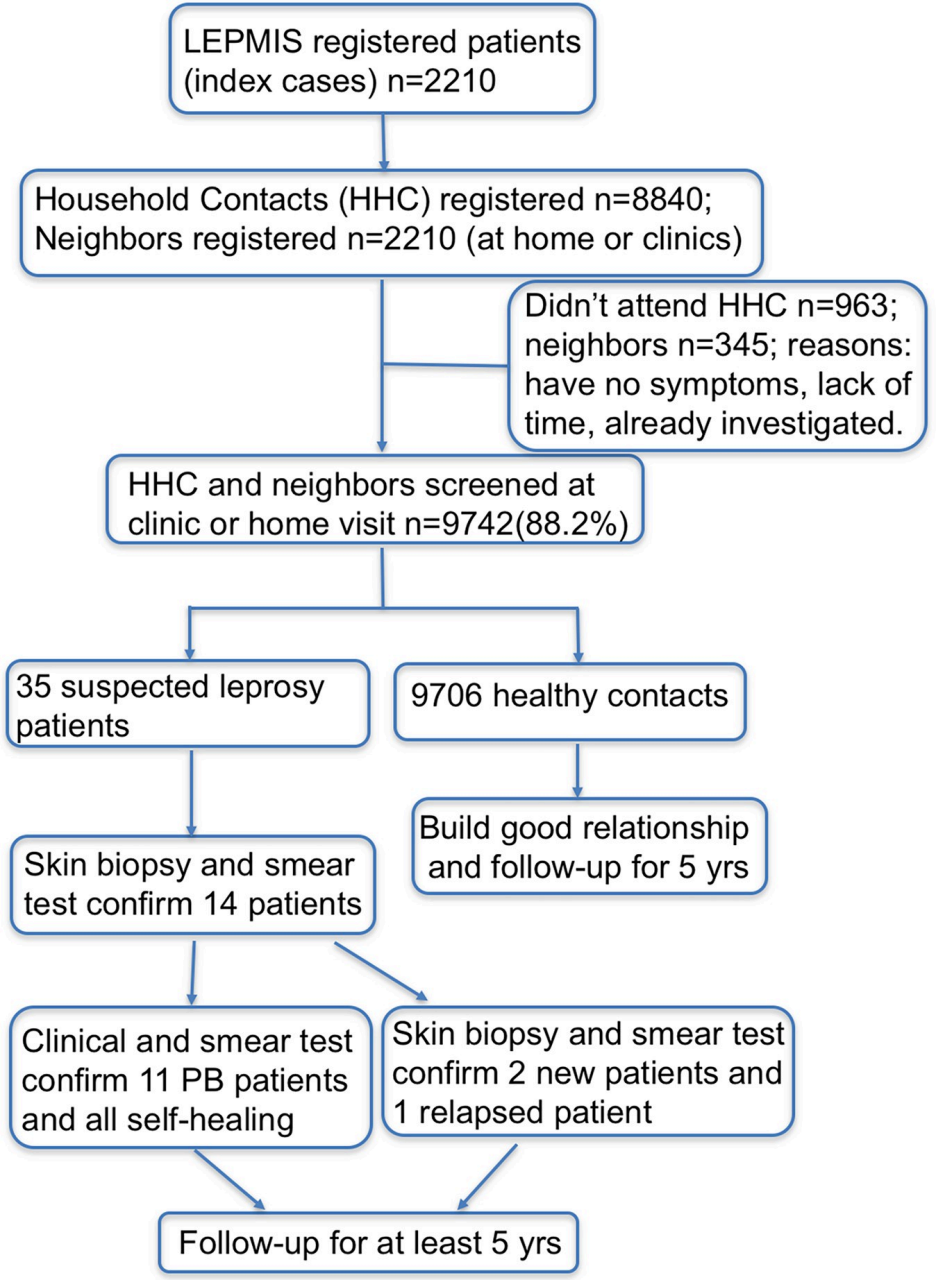
Flow chart of ACF program for patients with leprosy released from treatment and their contacts in Shandong Province, P.R. China.

All patients' medical data were anonymized and double-checked to ensure accuracy. An ACF flowchart is shown in Fig 1. All the questionnaire responses and patient data were collected and stored in Microsoft Excel 2010 (Microsoft Corporation, Redmond, Washington, USA). Pearson's chi-square test or Fisher's exact tests were used to analyze the categorical variables. The significance level was 0.05, and the confidence interval was 95%. Data management and analysis was performed using STATISTICA software (release 6.1, StatSoft, TIBCO Software Inc., Palo Alto, CA, USA).

Descriptive statistics were used to summarize key RFT and contacts data with the aims of investigating the feasibility of the survey, describing the main characteristics of the index patients and newly-detected leprosy patients among their contacts, and evaluating the effectiveness of ACF surveying as compared to passive case detection in Shandong Province. The feasibility of the survey was assessed through (i) the number of HHCs and NCs that were successfully screened; (ii) the number of new leprosy patients detected among the contacts, stratified by HHCs and NCs; (iii) the delay period of the new leprosy patients among contacts; and the total number of newly-detected leprosy patients detected through passive case detection.

Standard demographic and leprosy-specific variables were used to characterize the RFT patients and newly-detected leprosy patients among their contacts: sex (male/female), age group (≤14 years, 15–45, and ≥45 years of age), type of contacts, visible deformity, and history of leprosy in the family.

### Ethics statement

The study was approved by the Institutional Review Board at the Shandong Provincial Institute of Dermatology and Venereology. Written informed consent were obtained from all participants (or their guardians, if under 14 years old).

## Results

A total of 2,210 RFT patients and 9,742 contacts comprising 7,877 HHCs (including 5,844 genetic related family members (GRFMs) and 2033 non-genetic related family members) and 1,865 NCs were recruited. Demographic profiles of all RFT patients including age, gender, G2D, and family history information are in Table 1. The 2,210 RFT patients were from 2,102 families. The ratio of males to females was 3.58 and the mean age was 56.8 years. Among these patients, 104 RFT patients had a family history of leprosy (4.95%) and 58 families had multiple leprosy patients (mean: 3.1 patients, range: 2–5 patients per family).

**Table 1:** Sociodemographic and clinical characteristics of patients with leprosy released from treatment (n = 2210) in Shandong Province, P.R. China.

Characteristic	No. of patients	%
Age (years)		
0–14	13	0.59
15–45	50	2.26
≥45	2147	97.15
Sex		
Male	1727	78.14
Female	483	21.86
Type of contact		
GRFMs	5844	60.00
None- GRFMs	2033	20.86
NC	1865	19.14
Visible deformity		
Yes	1646	76.6
No	564	23.4
Follow-up patients		
Suspected cases	35	0.30
Confirmed cases	14	0.12
History of leprosy in family		
Yes	58	3.1
No	1727	96.9

GRFMs: genetic related family members; NC: neighbor contact; ACF: active case finding.

Index cases were predominantly male (78.1%) and 45 years of age or older (97%). No difference was seen in the gender distribution of HHCs and NCs (P = 0.1068). Mean age was higher among index cases (71.82 years) than among HHCs (50.56 years; P < 0.0001) or NCs (58.63 years; P < 0.0001).

Based on dermatological and neurological examinations, 35 people were initially suspected of having leprosy among 9,742 contacts without a personal history of leprosy. Diagnoses were confirmed for 13 new leprosy patients and one who relapsed based on clinical and histopathological examinations for a detection rate of 0.12%. Among 7,877 HHCs, nine had leprosy, in which one was from non-GRFMs; among 1,865 NCs, four were diagnosed as having leprosy, resulting in no difference in detection rates (chi-square test 0.51, *P* = 0.475 for HHCs and NCs). The patient who relapsed was a 50-year-old man who had been diagnosed with MB leprosy 24 years ago and was considered cured in November 1997. During our campaign survey in November 2014, this patient presented with multiple, infiltrated, erythematous plaques with mild pruritus on the hips that had been present for 5 months.

Of the 14 Chinese Han newly diagnosed patients with leprosy, three had MB leprosy and 11 had paucibacillary leprosy. Six patients had grade 1 disability and five of the 14 had G2D, giving a rate of 35.7%. Three patients had no disabilities. The average delay period for these 14 patients was more than three years. Among the 14 patients, nine (64.3%) had a family history of leprosy. All patients with new diagnoses received treatment for standard multidrug therapy (MDT). Dermatologic information and clinical characteristics of the 13 patients with new diagnoses and the patient who relapsed are shown in Table 2.

**Table 2:** Sociodemographic information for newly diagnosed patients with leprosy in Shandong Province, P.R. China (2014–2016).

Patients	Age(year)	Sex	G2D	*Delay in diagnosis (months)	Contact type	Leprosy type
Patient 1	66	female	No	60	non-GRFM	MB
Patient 2	76	male	Yes	18	GRFM	MB
Patient 3	50	male	Yes	5	relapse	MB
Patient 4	46	female	No	30	NC	PB
Patient 5	58	male	Yes	120	GRFM	PB
Patient 6	67	female	No	36	GRFM	PB
Patient 7	46	male	No	25	NC	PB
Patient 8	55	female	No	60	GRFM	PB
Patient 9	48	female	No	66	NC	PB
Patient 10	52	male	Yes	25	GRFM	PB
Patient 11	69	male	No	28	GRFM	PB
Patient 12	72	female	Yes	32	GRFM	PB
Patient 13	59	male	No	42	NC	PB
Patient 14	64	male	No	39	GRFM	PB

GRFMs: genetic related family members; NC: neighbor contact

*Delay in diagnosis: referred the patients who had the clinical manifestations until he came to clinic for the diagnosis.

## Discussion

This study used data from 2,210 RFT patients and their 9,742 contacts from 21 low-endemic counties in Shandong province of northern China. We identified 13 new patients with leprosy and one with relapse, which yielded a detection rate of 0.12%. In the same period, passive case detection in Shandong Province registered 15 new and relapsed patients. Thus, this ACF program yielded 48.3% (14/29) of all newly registered patients, indicating that ACF is an important way to improve detection in low-endemic areas. Furthermore, the ACF strategy could facilitate removal of potential sources of infection. This could reduce transmission to contacts and lead to early diagnosis of latent patients, reducing the incidence of G2D among contacts over time.

ACF for leprosy has been implemented in many countries and districts, such as Cambodia and Brazil. ACF is ideally carried out with the help of local staff who can readily identify and approach contacts, examine them, and refer those suspected of being infected for early confirmatory diagnosis. The experience with ACF among RFT and their contact individuals in Shandong province of China over the last years clearly demonstrates that an ACF approach is feasible and contributes to early case detection as indicated by the higher proportion of patients with PB leprosy diagnosed during the ACF compared to the respective proportion in routine passive case detection. However, case finding, whether active or passive, can identify only a certain fraction of all patients with leprosy [1]. Leprosy control cannot be solely based on ACF since the tracking of index cases and screening of HHC and NC individuals critically depends on a functioning passive case-detection system. That is, the basic capacity to diagnose leprosy must be maintained in the health control system. The drive to interrupt *M*. *leprae* transmission and eliminate leprosy is entering a crucial stage. ACF and passive case-detection strategies should not be compared competitively, but rather need to be interpreted as mutually enhancing for effective early detection of leprosy, which in turn can reduce G2D.

Over the last decade, the number of new patients with leprosy with G2D has remained constant, with a range of 12,437 to 14,409 worldwide, indicating stagnation in leprosy control [3]. In endemic areas such as Brazil, the proportion of new cases detected with G2D increased from 7.5% in 2015 to 8.3% in 2017 [18]. Facing this urgent situation, the WHO launched a 5-year global leprosy strategy in 2016, one aim of which was to reduce the number of new leprosy cases with G2D to < 1 per million people [19]. The results of our survey showed that patients with previously undetected disease are at high risk of G2D (35.7%), indicating insufficient HHC tracking and leprosy education among the population. Shandong Province (prevalence < 1/100,000) declared leprosy elimination as a public health problem in 1994. Following this milestone, leprosy control was gradually halted and the Leprosy Elimination Program atrophied. As a result, the G2D rate and family history among newly-diagnosed patients are stable or even increasing. Thus, exploring an appropriate tool to detect new patients with leprosy is urgently needed.

Several strategies for detecting early-stage leprosy were adopted to contribute to the aim of achieving early diagnosis and reducing the risk of disability in patients with leprosy and curbing the transmission of *M*. *leprae* [20–22]. In declared leprosy-elimination countries, passive case detection and integration of leprosy services into the general health system have become standard [23]. However, the fraction of patients with paucibacillary leprosy (PB) is not easy to detect passively. In a study of post-exposure prophylaxis (PEP), a cluster randomized, double-blind, placebo-controlled trial in Bangladesh gave a single dose of rifampicin to contacts of patients with leprosy, reducing leprosy incidence among contacts by 57% (95% confidence interval [CI] 33–72) in the first 2 years of the study [24]. Although a single dose of Rifampicin (SDR)-PEP is now recommended by WHO [25], in the low endemic area of Shandong province based on the previous report [24] and the leprosy risk prediction model [26], it is would not be a worthwhile addition for the leprosy program to achieve a zero incidence of leprosy in Shandong. Vaccines are generally seen as essential for eliminating a transmissible disease. Systematic reviews and meta-analyses suggest that the BCG vaccine has a protective efficacy of around 50% against leprosy, with greater protection against multibacillary than paucibacillary leprosy [27,28]. However, efficacious vaccines specifically targeting *M*. *leprae* remain elusive [3].

We saw clusters of patients with leprosy in family groups, with up to six patients in a single family. This phenomenon has also been reported in Indonesia [29]. Hastings RC group has described increasing family clustering as the endemicity level reduces since 1994 [30], which means in the low endemic area of Shandong province, surveillance of the RFT patients and their contact individuals is much more important. Numerous studies show that individuals living with a patient with untreated leprosy have a higher risk of developing clinical disease [6,31]. A 25-year follow-up research study in Sulawesi, Indonesia showed that 28 of 101 (28%) newly diagnosed patients were identified as household contacts and 36 (36%) identified as neighbors [8]. Mohanty et al. confirmed that transmission of *M*. *leprae* occurs among household members and is transmitted from social and neighborhood contacts in long-term association with patients with leprosy [32]. Thus, ACF can be an important early detection tool for tracking HHCs and NCs of patients with leprosy in a country with strategic targets for leprosy control. Our ACF program confirmed that implementing this strategy is necessary among HHCs and neighbors of RFT patients in historically high-endemic areas. Since leprosy has the initially painless and inconspicuous symptoms which can easily be mistaken for other skin conditions, our program also spread health education among contacts, who may be alerted to the risk of leprosy before they first encounter incipient symptoms.

### Limitations

A major limitation of this study was the use of non randomly sample groups obtained at the reference LEPMIS leprosy center. Firstly, the RFT patient from different regions based on the historically high endemic areas. Secondly, in the same region, the patients have need to informed consent, which limited us to randomly selected patients and their contact individuals, maybe this is one reason why our index cases were predominantly male. In addition, the sample group may have had selection bias toward older individuals, as children did not cooperate or their parents or guardians refused to let them be examined. However, the group in this study included contacts with a wide range of social and demographic characteristics who lived with low-income, which is common in China. Another limitation is the tracking of index cases and screening of their contact individuals critically depends on a functioning passive-case detection system, that does not compete with ACF, but is interpreted as mutually enhancing. Therefore, the economic benefit of our ACF is not easy to identify. The last but not least limitation is the delay in diagnosis of newly-detected leprosy patients, meaning that contact individuals were partially based on verbal screening, and contacts might have forgotten or not felt comfortable to report their own disease history before other relatives.

### Conclusion

Our ACF program demonstrates that our ACF approach is feasible and contributes to early case detection as indicated by the higher proportion of patients with PB leprosy diagnosed during the ACF compared to the respective proportion in the routine passive case detection. ACF and passive case-detection strategies should not be compared competitively, but rather need to be interpreted as mutually enhancing for effective early detection of leprosy, which in turn can reduce G2D.

## Supporting information

S1 Checklist STROBE checklist. (DOC)

S1 Fig a &b. a): show the location of Shandong province and marked with #. b): The 21 counties of Shandong Province were listed on the map, marked with red color names, the No. of * represent the No. of newly diagnosed patients in different counties. (maps from PlaniGlobe, http://www.planiglobe.com, CC BY 2.0).
(ZIP)

S1 Text Ethical approval for this study. (PDF)
