# Supplementary figures and images for: The role of an active surveillance strategy of targeting household and neighborhood contacts related to leprosy cases released from treatment in a low-endemic area of China
Source: PLoS Negl Trop Dis. 2020 Aug 14;14(8):e0008563. doi: 10.1371/journal.pntd.0008563 (PMC7485864; doi:10.1371/journal.pntd.0008563)

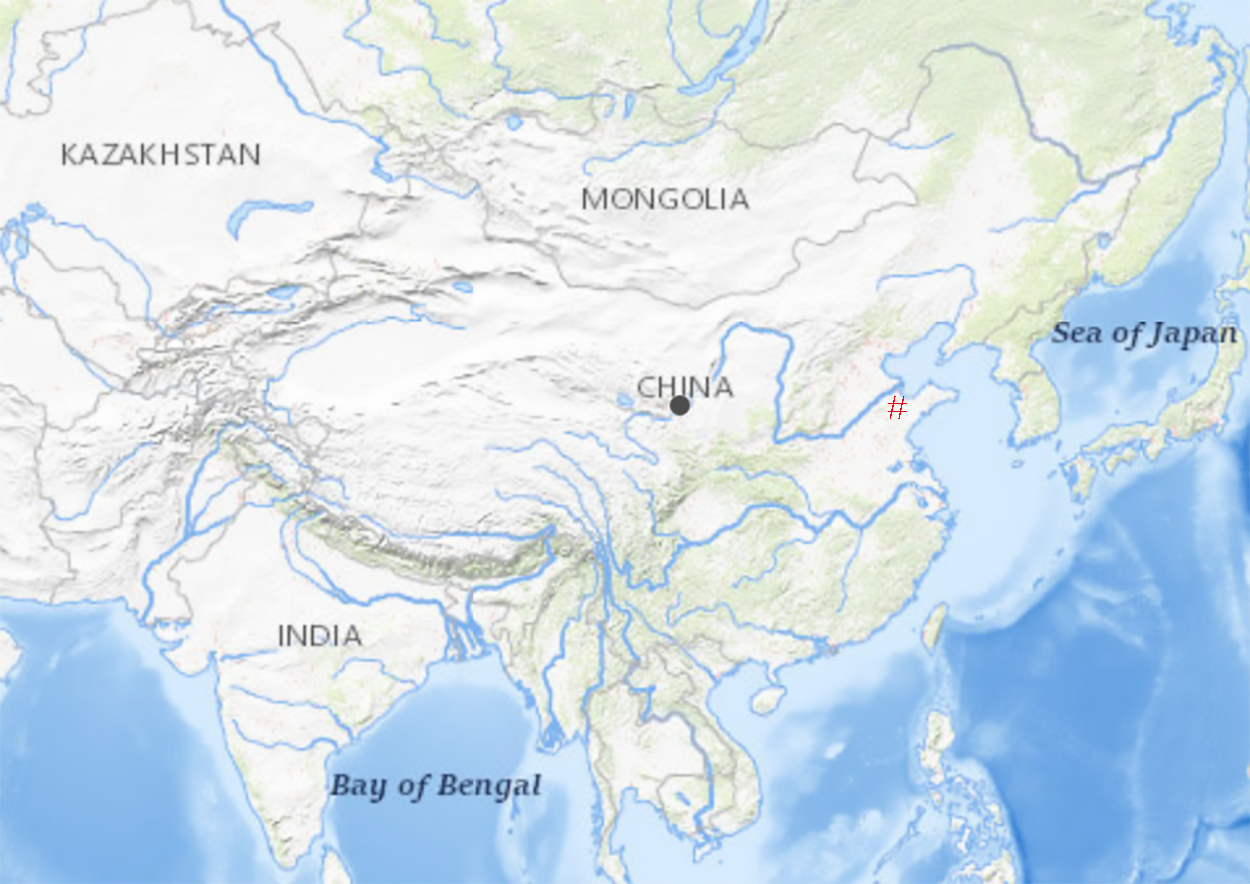

Supplement: S1 Fig — a &b. a): show the location of Shandong province and marked with #. b): The 21 counties of Shandong Province were listed on the map, marked with red color names, the No. of * represent the No. of newly diagnosed patients in different counties. (maps from PlaniGlobe, http://www.planiglobe.com, CC BY 2.0). (ZIP) [file pntd.0008563.s002.zip › S1 Fig/S1 Fig a.tif]

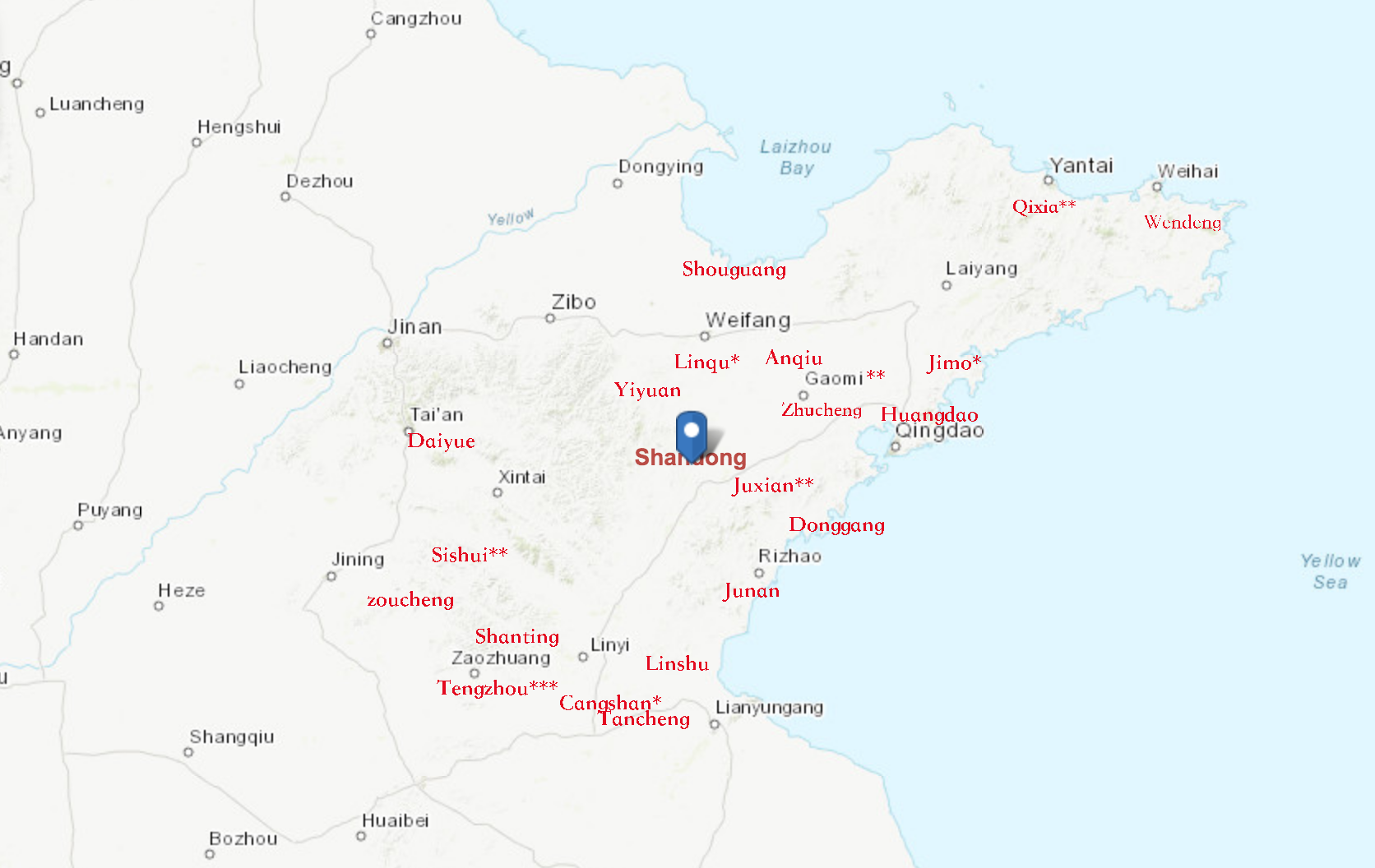

Supplement: S1 Fig — a &b. a): show the location of Shandong province and marked with #. b): The 21 counties of Shandong Province were listed on the map, marked with red color names, the No. of * represent the No. of newly diagnosed patients in different counties. (maps from PlaniGlobe, http://www.planiglobe.com, CC BY 2.0). (ZIP) [file pntd.0008563.s002.zip › S1 Fig/S1 Fig b.tif]
