# Supplementary material for: The role of an active surveillance strategy of targeting household and neighborhood contacts related to leprosy cases released from treatment in a low-endemic area of China
Source: PLoS Negl Trop Dis. 2020 Aug 14;14(8):e0008563. doi: 10.1371/journal.pntd.0008563 (PMC7485864; doi:10.1371/journal.pntd.0008563)
Supplement: S1 Text — (PDF) [file pntd.0008563.s003.pdf]

山东省皮肤病医院医学伦理委员会
临床研究审批表

编号：2014-019

项目名称	项目县麻风密切接触者筛查及风险模型的构建		
实验目的	为落实《山东省消除麻风危害规划(2011-2020年)》(鲁卫疾控字[2012]27号)的要求，减少乃至消除麻风所导致的畸残，山东省卫计委批准，在山东省选择麻风高发县区（历史累计病例超过500的21个重点县(市、区)开展麻风病例早期发现项目，探索在农村/社区通过开展麻风病密切接触者检查和疑似病例转诊主动发现麻风病例的有效模式。重新评价家庭内密切接触者筛查的必要性，为早期发现麻风患者，减少畸残发生，提供更强有力的证据。<br>项目组前期已开展了系列的麻风遗传学研究，建立了包括350个家系和10000个散发病例及与之相匹配的近15,000例正常对照的遗传资源库，并利用遗传资源库中麻风散发病例和正常对照开展了GWAS，先后定位了麻风的25个风险位点。研究结果发表在New England Journal of Medicine、Nature Genetics等杂志上。本项目拟在此基础上，基于已定位的25个风险位点研发高危个体易感基因筛查芯片，构建麻风发病的风险预测模型，以期能筛查麻风高危人群、亚临床患者的确定提供依据，为实现麻风的一级预防，消除麻风危害奠定基础。		
申请单位	山东省皮肤病性病防治研究所暨山东省皮肤病医院		
申请人	张福仁		
预计时间	2014.08-2018.07		
申请日期	2014-2-17	开会日期	2014-2-24
伦理委员会成员列表	王　婷　田洪青　田仁明　温绪宗<br>张　华　仲丽华　冯国禄		
本次伦理会议出席人员	王　婷　田洪青　田仁明　温绪宗<br>张　华　仲丽华　冯国禄		
伦理委员会成员出席人数：7人	回避人数：0人	弃权人数：0人	
讨论结果　同意：7票	不同意：0票	作必要修改后同意：0票	作必要修改后重新审核：0票

伦理委员会评审意见：

通过对申请人陈述“项目县麻风密切接触者筛查及风险模型的构建”课题的背景、研究方案、涉及伦理问题的讨论和投票表决，伦理委员会认为该研究方案设计合理，保证了研究对象的的权益，同意该研究项目。提醒研究者应该清楚医学伦理规范，按照伦理对反和方案开展研究，保护研究对象的权益。

主任委员签名：王婷

山东省皮肤病医院医学伦理委员会盖章

（印章：山东省皮肤病医院 医学伦理委员会）

2014年02月24日

本审批表一式三份，申请人、专业科室和山东省皮肤病医院医学伦理委员会各存一份。

Ethical review and approval documents made by Ethics Committee of Shandong Provincial Hospital for Skin Disease

No.	2014-019		
Project name	**An Active Case-Finding Program Targeting Household and Neighborhood Contacts in a Low-Endemic Area of China**		
Project research unit	Shandong Provincial Institute of Dermatology and Venereology<br>Shandong Provincial Hospital for Skin Disease		
Name of applicant	Furen Zhang		
The main content of studies involving the human	Shandong achieved the elimination of leprosy in 1994; however, the district continues to report a relatively high number of cases of leprosy infection involving deformity. Several studies have shown that individuals in contact with those who are infected with leprosy are at high risk of developing the disease, and subclinical infections among such individuals are important in the chain of *Mycobacterium leprae* transmission. Some hyperendemic areas have shown growing interest in active case finding. Furthermore, recent data from the World Health Organization indicate high rates of relapse as well as extensive family history of leprosy among people in the Shandong region of P.R. China, indicating a need to reconsider the current approach to prevention. Active case finding was conducted in 20 counties of Shandong Province among people infected with leprosy and their contacts. Investigators and subjects have no conflicts of interest. The subjects' personal privacy is protected.		
Application date	2014-02-17	Date of a conference	2014-02-24
Attendees	Member of EC: 7	Member of Presented: 7	Member of Parries: 0
Opinion	Member of approved: 7	The necessary corrections for Approved: 0	
	Mem ber of Rejected: 0	The necessary modifications for retrial: 0	

Review comment:

This study is planned according to the ministry of health "involves people of biomedical research ethics review method (trial) (2007)", and will be performed in accordance with the ethical standards laid down in the 1964 Declaration of Helsinki. In this study, subjects' rights and interests are fully protected. There is no potential risk to the subjects. The board of ethics committee agreed to the study work as planned.

The chairman of ethics committee signed: Ting Wang

Seal:

Date: 2014.02.24

Note: This form shall be submitted by the Scientific Research Division to the EC office of the hospital.

Ethical review and approval documents made by Ethics Committee of Shandong Provincial Hospital for Skin Disease

<table>
<tr><td>No.</td><td colspan="6">2014-019</td></tr>
<tr><td>Project name</td><td colspan="6">The development and application of the leprosy risk screening chip and risk assessment for House-hold contacts and the people infected with leprosy</td></tr>
<tr><td>Project research unit</td><td colspan="6">Shandong Provincial Institute of Dermatology and Venereology
Shandong Provincial Hospital for Skin Disease</td></tr>
<tr><td>Name of applicant</td><td colspan="6">Furen Zhang</td></tr>
<tr><td>The main content of studies involving the human</td><td colspan="6">Our research team previously have performed a series of genetic investigations for leprosy , with the fund of the grant from National Nature Science foundation of China(30771943) and Shandong Science and technology research projects (2006GG2302029), we have built the genetic resources library, including the 350 leprosy pedigrees, 10000 sporadic leprosy patients and 15,000 healthy control. Based on this library we performed the genome wide association studies (GWAS) and totally identified 25 susceptibility loci, which have been published on the journals of New England Journal of Medicine, Nature Genetics, et al. According to the previous finding, we constructed the leprosy screening chip with the 25 susceptibility loci, in order to screen leprosy high-risk population and provide effective method for early diagnosis of subclinical patients. This risk screening chip in some extent can lay the foundation for primary prevention and eliminate the harm of leprosy. Investigators and subjects have no conflicts of interest. The subjects' personal privacy is protected.</td></tr>
<tr><td>Application date</td><td colspan="3">2014-02-17</td><td colspan="2">Date of a conference</td><td>2014-02-24</td></tr>
<tr><td>Attendees</td><td>Member of EC: 7</td><td colspan="3">Member of Presented: 7</td><td colspan="2">Member of Parries: 0</td></tr>
<tr><td rowspan="2">Opinion</td><td colspan="2">Member of approved: 7</td><td colspan="4">The necessary corrections for Approved: 0</td></tr>
<tr><td colspan="2">Mem ber of Rejected: 0</td><td colspan="4">The necessary modifications for retrial: 0</td></tr>
</table>

Review comment:

This study is planned according to the ministry of health "involves people of biomedical research ethics review method (trial) (2007)", and will be performed in accordance with the ethical standards laid down in the 1964 Declaration of Helsinki. In this study, subjects' rights and interests are fully protected. There is no potential risk to the subjects. The board of ethics committee agreed to the study work as planned.

The chairman of ethics committee signed: Ting Wang

Seal:

Date: 2014.02.24

Note: This form shall be submitted by the Scientific Research Division to the EC office of the hospital.
